# Association between the triglyceride-glucose (TyG) index and stroke risk in Chinese normal-weight adults: a population-based study

**DOI:** 10.1186/s13098-024-01421-w

**Published:** 2024-07-25

**Authors:** Man Wu, Chaoyang Li, Yiqing Yu, Lijuan Zeng, Yufei Qiu, Jiali Liu, Fen Yang, Yangyang Han

**Affiliations:** 1 Chengdu Third People’s Hospital, Chengdu, China; 2 School of Nursing, Hubei University of Chinese Medicine, Wuhan, China; 3 Zhongnan Hospital of Wuhan University, Wuhan, China; 4 Hubei Shizhen Laboratory, Wuhan, China; 5 School of Nursing, Shandong First Medical University & Shandong Academy of Medical Sciences, Taian, China

**Keywords:** TyG index, Stroke, Normal-weight, Risk factor

## Abstract

**Background:**

Identifying high-risk populations and promoting stroke prevention measures can be achieved through studies on stroke and its risk factors. As a new alternative indicator of insulin resistance (IR), the triglyceride glucose (TyG) index may potentially increase stroke risk. However, the evidence confirming this association is inadequate and inconsistent, possibly due to variations in stroke assessment criteria or characteristics of the study populations. This study aims to evaluate the association between the TyG index and stroke risk level among individuals with normal-weight.

**Methods:**

A total of 30,895 participants aged ≥ 40 years with normal-weight were enrolled in this study. The TyG index was calculated as Ln [fasting triglycerides (mg/dL) × fasting glucose (mg/dL)/2]. Normal-weight was described as a body mass index (BMI) of 18.5-<24.0 kg/m^2^. Stroke risk was assessed by the Stroke Risk Assessment Scale, developed by the China National Stroke Screening and Prevention Project. To evaluate the associations between the TyG index and stroke risk level, multivariate logistic regression models were employed.

**Results:**

Results showed that when the TyG index was considered as a continuous variable, each one unit increase in the TyG index was associated with a significantly higher risk of stroke [Moderate-risk (OR, 2.15; 95% CI, 2.03–2.28; *P*<0.001); High-risk (OR, 3.83; 95% CI, 3.57–4.10; *P*<0.001)]. Compared with Q1 of the TyG index, Q4 was significantly associated with moderate stroke risk (OR, 2.73; 95% CI, 2.50–2.99; *P*<0.001) and high stroke risk (OR, 5.39; 95% CI, 4.83–6.01; *P*<0.001). The continuous TyG index was an important risk factor for high stroke risk in the metabolically obese, normal-weight (MONW) individuals (OR, 3.44;95% CI, 2.92–4.06; *P* < 0.001). In the MONW individuals, when Q1 was used as a reference, participants in Q4 (OR, 5.33; 95% CI, 4.19–6.78; *P* < 0.001) was significantly associated with high stroke risk. Subgroup analysis showed significant interaction in the age and sex subgroups in the overall population (*P*_interaction_ <0.001).

**Conclusion:**

The risk of stroke is increased with the TyG index among Chinese adults of normal weight; hence, the index may be an important indicator for identifying high-risk stroke populations among individuals with normal body weight.

**Supplementary Information:**

The online version contains supplementary material available at 10.1186/s13098-024-01421-w.

## Introduction

Stroke is one of the most common causes of disability and death worldwide [1]. Age-standardized stroke incidence significantly decreased as a result of improved healthcare services and the deployment of preventative interventions aimed at cerebrovascular risk factors. However, the situation in developing countries is considerably different [2]. Based on findings from the National Health Commission of Stroke Screening and Prevention Projects (NHCSSPP) in China, roughly 20% of individuals aged 40 and older are identified as being at high risk for stroke [3]. Critically impairing quality of life and imposing substantial economic and social burdens are all consequences of stroke [4]. While risk factors for stroke, such as hypertension, diabetes, and obesity, have been extensively studied [5]; however, available evidence suggests that metabolic abnormalities may contribute to an increased risk of stroke even among normal-weight individuals [6]. Some individuals with normal-weight, referred to as MONW, may exhibit notable metabolic issues, including insulin resistance, lipid abnormalities, and elevated blood pressure [7]. The lack of overweight or obesity in these individuals may render them undetectable, potentially resulting in missed opportunities to benefit from suitable intervention strategies [8]. Hence, an immediate requirement exists for readily available and dependable biomarkers that can aid in the early detection of moderate to high stroke risk in individuals with normal-weight.

The triglyceride-glucose (TyG) index, recognized as a straightforward and cost-effective biomarker of insulin resistance (IR) and overall metabolic health, has garnered growing attention in recent years [9]. From venous blood extraction levels of triglyceride and glucose, the TyG index is computed (fasting triglyceride [mg/dL] × fasting glucose [mg/dL]/2) [10]. The TyG index has been confirmed to be a dependable substitute for IR due to its simplicity and cost-effectiveness [9]. IR has been determined as an important risk factor for stroke occurrence and development [11]. The results currently accessible indicate that the TyG index is a better predictor of stroke than the Homeostasis Model Assessment (HOMA-IR) [12]. The TyG index shows an association with the risk of stroke, albeit among various study populations [13]. Studies have demonstrated that the TyG index can identify individuals with a higher risk of IR and cardiovascular disease (CVD) among otherwise healthy populations [14]. Regarding the normal-weight Chinese population, the TyG index has been confirmed to be significantly associated with the risk of developing type 2 diabetes mellitus (T2DM) [15]. Several studies have investigated the ability of the TyG index to predict the occurrence of T2DM [16] and CV [17] events in apparently healthy populations. Similarly, research has indicated that the TyG index is effective in identifying certain CVD risk factors and individuals at high risk for MONW [14]. Undoubtedly, the TyG index has attracted considerable interest for its role in analyzing variations in predicting the risk of CVD. Although initial research has provided vital information, there is still a substantial lack of understanding regarding the correlation between the TyG index and the risk of stroke, especially among different populations.

Research on whether the TyG index is a reliable indicator of stroke risk in people with normal weight is currently lacking. It is necessary to conduct stroke risk stratification among individuals with normal-weight, specifically pinpointing those at moderate to high risk of stroke.

## Methods

### Study design and participants

**Fig. 1:**
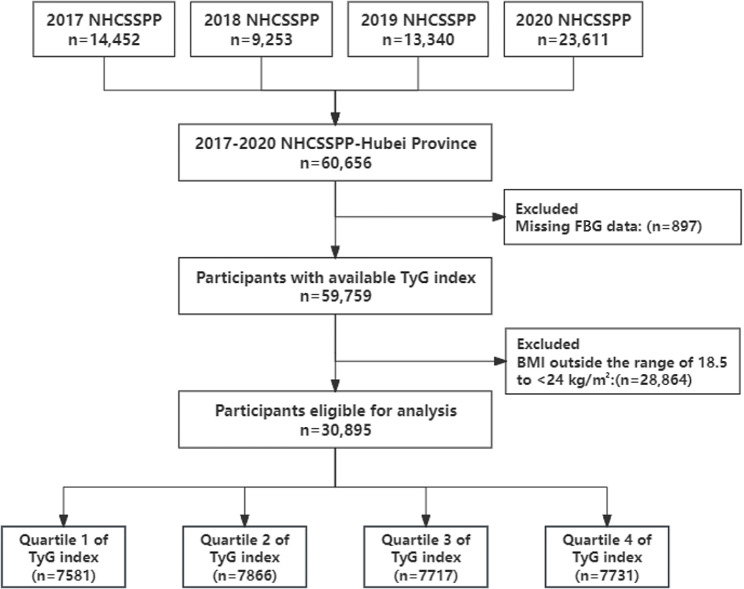
Flowchart of subject selection in the study

Participants were recruited via the Stroke Screening and Prevention Project in Hubei Province, China, which ran from 2017 to 2020. A cluster sampling method was used to select the samples. Choose 5 cities and 9 communities depending on the ratio of local population size and community size, and carry out interviews with all inhabitants aged 40 or older during the primary screening phase. Performing questionnaire surveys, physical examinations, and assessing stroke risk factors at primary healthcare institutions. However, participants were excluded if they were seriously unwell and were unable to complete questionnaires or physical examinations. A total of 60,656 participants were included in this study, excluding 897 participants who lacked fasting blood glucose (FBG) data and 28,864 participants whose BMI did not fall within the range of 18.5 to < 24 kg/m^2^. Finally, 30,895 participants were eventually included in the statistical analysis **(**Fig. 1**)**.

### Measurements and definitions

Trained surveyors collected fundamental data from participants through the use of questionnaires. Sociostatistical characteristics include age, sex, marital status, education level, physical activity, personal average annual income, smoking and drinking, cerebrovascular disease, hypertension, dyslipidemia, diabetes, and other disease history. Drinking is defined as current alcohol consumption or not drinking (including never drinking and quitting alcohol). The physical examination involves the assessment of weight, height, waist circumference (WC), and blood pressure (BP). BMI was calculated as body weight divided by the square of height (kg/m^2^). BP is the average of three readings taken at 1-minute intervals after a 5-minute respite. All participants collected blood samples after fasting for at least 8 h. Triglyceride (TG), total cholesterol (TC), FBG, low-density lipoprotein cholesterol (LDL-C), high-density lipoprotein cholesterol (HDL-C), and other indicators were determined from blood samples.

The Stroke Risk Assessment Scale was established by the Stroke Screening and Prevention Project Committee, National Health and Family Planning Commission of China [18]. Three risk level evaluation criteria and eight stroke risk factors comprise this scale (hypertension, lipid profile, diabetes, atrial fibrillation, smoking, obesity, lack of exercise, and family history of stroke). Among them, hypertension is described as having a previous diagnosis of hypertension, using antihypertensive medicines, and/or the average of two on-site measures of the systolic blood pressure (SBP) ≥ 140mmHg and/or diastolic blood pressure (DBP) ≥ 90 mmHg at rest. Atrial fibrillation may be diagnosed using a typical 12-lead ECG. Diabetes is defined as previous diagnosis, and/or the use of insulin or oral hypoglycemic medicine for treatment. Dyslipidaemia is characterized by the use of antilipidaemic medicine or the presence of one or more of the following conditions: TC ≥ 5.18 mmol/L, TG ≥ 1.70 mmol/L, or LDL-C ≥ 3.10 mmol/L. Smoking is defined as current smoking or non-smoking (including never smoking and quitting). Minimal or mild physical activity (< 3 times a week, < 30 min each session, lasting for or 1 year) is considered a lack of exercise. A BMI greater than or equal to 26 kg/m² classifies an individual as overweight or obese. The stroke family history is confined to direct relatives. Participants in the low-risk stroke population have less than 3 out of 8 risk factors and do not have any chronic diseases. The moderate-risk stroke group consists of individuals who have less than 3 out of 8 risk factors and have at least one of the 3 chronic conditions, namely hypertension, diabetes, and atrial fibrillation. Individuals who possess three or more of the eight risk factors or have a history of stroke or transient ischemic attack (TIA), are considered to be at high risk for stroke.

The diagnostic criteria for MONW individuals need them to fulfill the diagnosis of metabolic syndrome (MetS) while having a normal body weight. Normal-weight was described as BMI ranging between 18.5 to less than 24 kg/m^2^ [19]. The diagnostic criteria for MetS [20] in China are as follows: (1) Central obesity (WC): males ≥ 90 cm, females ≥ 85 cm; (2) Hypertension: BP ≥ 130/85 mmHg, or use of antihypertensive drug therapy; (3) Hypertriglyceridemia: TG ≥ 1.7 mmol/L; (4) HDL-C: males < 1.03 mmol/L, females < 1.29 mmol/L; (5) Hyperglycemia: FBG ≥ 5.6 mmol/L or previous medical diagnosis. A diagnosis can be established provided that three of the mentioned five criteria are met. Otherwise, it is considered metabolically healthy with normal-weight (MHNW).

### Statistical analysis

All analyses were conducted using SPSS 26.0 and RStudio 4.3.2. Categorical variables are expressed as the number or percentage, and continuous variables are represented by mean ± standard deviation (M ± SD) or median and interquartile ranges (IQRs). To examine the linear trend of the TyG index quartiles among the basic characteristics of the study population, a linear trend chi-square test was used for categorical variables. For continuous variables with normal and skewed distributions, one-way analysis of variance (ANOVA) for linear trend and the Kruskal-Wallis test were employed, respectively. Using a multivariate logistic regression model with the lowest quartile as the reference, the odds ratio (OR) and 95% confidence interval (CI) of stroke risk by quartiles of TyG were calculated. Three logistic models were employed in this research: (a) adjust by variables including age and sex; (b) adjust by variables including age, sex, marital status, level of education, personal average annual income, and drinking; (c) adjust by variables including age and sex, marital status, level of education, personal average annual income, drinking, BMI, WC, SBP, DBP, TC, LDL-C, and HDL-C. In addition, stratified analyses were performed according to age (<60 and ≥ 60 years) and sex (male and female) to examine the consistency of the TyG index impact on stroke risk. A cross-product interaction term was added to the regression model and the Wald test was used to assess the statistical significance of the interaction. Subsequently, a restricted cubic spline regression model was employed to examine the relationship between TyG as a continuous variable and stroke risk in the overall population and MONW/MHNW population.

## Results

### Characteristics of the study population

Table 1 shows the basic characteristics of participants based on the quantiles of the TyG index. The average age of 30,895 participants was 59.48 (10.87) years old, of which 12,729 (41.20%) were male, 10,553 (34.16%) had a high school education or above, and 29,220 (94.58%) were married. Additionally, 3,480 (11.26%) were current smokers, 4,370 (14.14%) were current drinkers, and 20,833 (67.43%) frequently participated in physical activity. Compared to participants in the lowest quartile of the TyG index, those in the higher quartile tend to be older, male, less educated, lower-income, more frequently engage in physical activity, and currently consume alcohol. Except for marital status, drinking, atrial fibrillation, and previous TIA history, there were significant differences among the quartiles of the TyG index (*P*_for trend_ < 0.001). In addition, laboratory results, including SBP, DBP, TG, TC, FBG, LDL-C, HDL-C, etc., also showed significant differences between the quartiles of the TyG index (*P*_for trend_ < 0.001). Comparative analyses of demographic characteristics between enrolled and excluded subjects are shown in **Supplementary Table 1**.

**Table 1:** Comparison of characteristics according to the quartile of the TyG index

Characteristics	Overall (*n* = 30,895)	Quartile of TyG index				*P* for trend
		Quartile 1 (*n* = 7,581)	Quartile 2 (*n* = 7,866)	Quartile 3 (*n* = 7,717)	Quartile 4 (*n* = 7,731 )	
Range of TyG index	6.09–12.63	6.09–8.29	8.29–8.66	8.66–9.04	9.04–12.63	
Age(years)	59.48 ± 10.87	59.13 ± 11.19	59.18 ± 10.89	59.25 ± 10.67	60.63 ± 10.66	<0.001
Male, n (%)	12,729(41.20)	3065(40.43)	3192(40.58)	3167(41.04)	3305(42.75)	0.003
High school and above, n (%)	10,553(34.16)	2756(36.35)	2741(34.85)	2539(32.90)	2517(32.56)	0.001
Married, n (%)	29,220(94.58)	7118(93.89)	7472(94.99)	7338(95.09)	7292(94.32)	0.244
Personal average annual income ≥ 20,000 RMB, n (%)	17,102(55.36)	4219(55.65)	4427(56.28)	4366(56.58)	4090(52.90)	0.001
Current smoker, n (%)	3480(11.26)	1027(13.55)	845(10.74)	790(10.24)	818(10.58)	<0.001
Current drinker, n (%)	4370(14.14)	1122(14.80)	1028(13.07)	1096(14.20)	1124(14.54)	0.818
Physical activity, n (%)	20,833(67.43)	5281(69.66)	5308(67.48)	5055(65.50)	5189(67.12)	<0.001
Atrial fibrillation, n (%)	283(0.92%)	62(0.82%)	65(0.83%)	82(1.06%)	74(0.96%)	0.178
Family history of stroke, n (%)	2321(7.51%)	700(9.23%)	585(7.44%)	521(6.75)	515(6.66)	<0.001
Previous stroke history, n (%)	749(2.42%)	172(2.27%)	163(2.07%)	172(2.23%)	242(3.13)	<0.001
Previous TIA history, n (%)	391(1.27%)	90(1.19%)	95(1.21%)	101(1.31%)	105(1.36)	0.281
BMI, kg/m^2^	21.97 ± 1.34	21.73 ± 1.39	21.96 ± 1.34	22.09 ± 1.29	22.09 ± 1.29	<0.001
TyG index	8.68 ± 0.58	7.97 ± 0.25	8.48 ± 0.11	8.84 ± 0.11	9.43 ± 0.35	<0.001
WC, cm	79(74–83.00)	78(72–82)	79(74–83)	80(75–84)	80(76–85)	<0.001
SBP, mmHg	125.5(117.5–135)	125(115–136)	125(117–134)	125.5(118.5–134)	127(120–137)	<0.001
DBP, mmHg	77.5(72-82.5)	77(71-82.5)	77.5(72-82.5)	77.5(72.5–82.5)	78(73–83)	<0.001
TC, mmol/L	4.60(4.00-5.34)	4.30(3.65–4.98)	4.46(3.90–5.09)	4.70(4.13–5.33)	5.13(4.36–5.96)	<0.001
TG, mmol/L	1.37(1.00-1.89)	0.81(0.67–0.96)	1.23(1.10–1.36)	1.61(1.45–1.84)	2.34(1.97–3.10)	<0.001
FBG, mmol/L	5.08(4.56–5.79)	4.60(4.20–5.08)	4.90(4.50–5.30)	5.21(4.79–5.86)	5.82(5.10–7.97)	<0.001
LDL-C, mmol/L	2.57(2.09–3.16)	2.40(1.88–2.96)	2.58(2.12–3.10)	2.57(2.10–3.12)	2.77(2.13–3.50)	<0.001
HDL-C, mmol/L	1.40(1.17–1.69)	1.53(1.25–1.83)	1.41(1.20–1.68)	1.40(1.19–1.65)	1.28(1.04–1.55)	<0.001

*Abbreviations: TIA*, transient ischemic attack; *BMI*, body mass index; *WC*, waist circumference; *SBP*, systolic blood pressure; *DBP*, diastolic blood pressure; *TC*, total cholesterol; *TyG*, triglyceride glucose; *FBG*, fasting blood glucose; *HDL-C*, high-density lipoprotein cholesterol; *LDL-C*, low-density lipoprotein cholesterol

### Association between the TyG index and stroke risk

Among the 30,895 participants, 8,547 were classified as having a moderate risk of stroke, while 5,412 participants were classified as having a high risk of stroke. According to the quartile of the TyG index, the proportion of stroke risk in all four groups showed an upward trend. For moderate stroke risk, there were 1,965, 1,977, 2,120, and 2,485 cases in each quartile, respectively. For high stroke risk, there were 863, 959, 1,204, and 2,386 cases in each quartile, respectively. The results of multivariate logistic regression analysis are shown in Table 2. Overall, there was a significant positive association of the TyG index with stroke risk. The ORs of Moderate- and high stroke risk increased with the TyG index quartiles. After adjusting for age, sex, education level, marital status, personal average annual income, drinking, BMI, WC, SBP, DBP, TC, LDL-C, and HDL-C, when the TyG index was used as a continuous variable, each one unit increment of TyG index was significantly associated with an increase in moderate stroke risk (OR, 2.15; 95% CI, 2.03–2.28; *P*<0.001) and high stroke risk (OR, 3.83; 95% CI, 3.57–4.10; *P*<0.001). Specifically, in the categorical analyses, compared with Q1, participants in Q4 were significantly associated with moderate stroke risk (OR, 2.73; 95% CI, 2.50–2.99; *P*<0.001) and high stroke risk (OR, 5.39; 95% CI, 4.83–6.01; *P*<0.001).

**Table 2:** Associations between the TyG index quartiles with stroke risk

Stroke risk	Variables	Count (n)	Model 1		Model 2		Model 3	
			OR (95% CI)	*P*-value	OR (95% CI)	*P*-value	OR (95% CI)	*P*-value
**Moderate-risk** (***n*** = **8**,**547**)	Per 1 unit increase		1.75(1.67–1.84)	<0.001	1.77(1.68–1.85)	<0.001	2.15(2.03–2.28)	<0.001
	Q1	1,965	1.00 (ref.)		1.00 (ref.)		1.00 (ref.)	
	Q2	1,977	0.97(0.89–1.05)	0.409	0.98(0.91–1.05)	0.537	1.02(0.94–1.12)	0.595
	Q3	2,120	1.18(1.09–1.28)	<0.001	1.19(1.11–1.29)	<0.001	1.30(1.19–1.42)	<0.001
	Q4	2,485	2.15(1.99–2.32)	<0.001	2.18(2.02–2.35)	<0.001	2.73(2.50–2.99)	<0.001
**High-risk** (***n*** = **5**,**412**)	Per 1 unit increase		3.42(3.23–3.63)	<0.001	3.49(3.29–3.71)	<0.001	3.83(3.57–4.10)	<0.001
	Q1	863	1.00 (ref.)		1.00 (ref.)		1.00 (ref.)	
	Q2	959	1.08(0.98–1.20)	0.123	1.11(1.00-1.24)	0.045	1.10(0.98–1.24)	0.092
	Q3	1,204	1.57(1.42–1.74)	<0.001	1.62(1.46–1.79)	<0.001	1.60(1.43–1.79)	<0.001
	Q4	2,386	4.85(4.42–5.33)	<0.001	5.10(4.64–5.61)	<0.001	5.39(4.83–6.01)	<0.001

*OR* odds ratio, *CI* confidence interval

Model 1: adjusted for age, and sex

Model 2: adjusted for variables in Model 1 + marital status, education level, personal average annual income, drinking

Model 3: adjusted for variables in the Model 2 + BMI, WC, SBP, DBP, TC, LDL-C, HDL-C.

### Associations between the TyG index and stroke risk in different metabolic states of normal-weight

The association between TyG index and stroke risk according to different metabolic states with normal-weight (MONW/MHNW) is shown in Table 3. MONW and MHNW populations elevated ORs increased with quartiles of the TyG index. Specifically, after adjusting for age and sex, compared to Q1, MHNW participants in Q4 were significantly associated with moderate (OR, 1.60; 95% CI, 1.48–1.74; *P* < 0.001), and high (OR, 2.45; 95% CI, 2.19–2.73; *P* < 0.001) stroke risk respectively; and MONW participants in Q4 were significantly associated with moderate stroke risk (OR, 1.59; 95% CI, 1.29–1.97; *P* < 0.001) and high stroke risk (OR, 3.21; 95% CI, 2.61–3.95; *P* < 0.001) compared with Q1. Even after adjusting for education, marriage, personal average annual income, and drinking, participants with the TyG index Q4 were most significantly associated with high stroke risk in the MHNW (OR, 2.58; 95% CI, 2.31–2.89; *P* < 0.001) and MONW (OR, 3.33; 95% CI, 2.70–4.11; *P* < 0.001) populations, respectively. After adjusting for age, sex, education, marriage, personal average annual income, drinking, BMI, WC, SBP, DBP, TC, LDL-C, and HDL-C in model 3, when used as a continuous variable, the TyG index was an important risk factor for higher stroke risk in MONW individuals (OR, 3.44; 95% CI, 2.92–4.06; *P* < 0.001). In MONW participants, compared to Q1, participants in Q4 (OR, 5.33; 95% CI, 4.19–6.78; *P* < 0.001) was significantly associated with high stroke risk.

**Table 3:** Multinomial logistic regression model for the association of TyG index quartiles with stroke risk in MONW and MHNW states

Stroke risk												
	Moderate-risk						High-risk					
	Model 1 OR (95% CI)	*P*-value	Model 2 OR (95% CI)	*P*-value	Model 3 OR (95% CI)	*P*-value	Model 1 OR (95% CI)	*P*-value	Model 2 OR (95% CI)	*P*-value	Model 3 OR (95% CI)	*P*-value
**MHNW** (***n*** = **25**,**261**)												
**TyG index**	1.48(1.39–1.57)	<0.001	1.49(1.41–1.58)	<0.001	2.15(2.00-2.30)	<0.001	2.11(1.96–2.28)	<0.001	2.16(2.00-2.33)	<0.001	2.86(2.63–3.12)	<0.001
Q1	1.00 (ref.)		1.00 (ref.)		1.00 (ref.)		1.00 (ref.)		1.00 (ref.)		1.00 (ref.)	
Q2	0.97(0.89–1.06)	0.559	0.98(0.89–1.06)	0.613	1.04(0.94–1.14)	0.484	1.05(0.93–1.18)	0.413	1.08(0.96–1.22)	0.223	1.08(0.95–1.23)	0.239
Q3	0.97(0.89–1.06)	0.506	0.98(0.90–1.07)	0.673	1.16(1.05–1.28)	0.003	1.16(1.03–1.30)	0.014	1.19(1.07–1.35)	0.003	1.30(1.14–1.48)	<0.001
Q4	1.60(1.48–1.74)	<0.001	1.63(1.49–1.77)	<0.001	2.46(2.23–2.71)	<0.001	2.45(2.19–2.73)	<0.001	2.58(2.31–2.89)	<0.001	3.45(3.05–3.91)	<0.001
**MONW** (***n*** = **5**,**634**)												
**TyG index**	1.32(1.15–1.52)	<0.001	1.33(1.16–1.53)	<0.001	1.98(1.69–2.32)	<0.001	2.41(2.10–2.77)	<0.001	2.45(2.13–2.82)	<0.001	3.44(2.92–4.06)	<0.001
Q1	1.00 (ref.)		1.00 (ref.)		1.00 (ref.)		1.00 (ref.)		1.00 (ref.)		1.00 (ref.)	
Q2	0.66(0.55–0.79)	<0.001	0.67(0.56–0.81)	<0.001	0.72(0.59–0.88)	0.001	0.66(0.55–0.80)	<0.001	0.69(0.57–0.84)	<0.001	0.69(0.56–0.86)	0.001
Q3	0.92(0.77–1.12)	0.413	0.94(0.78–1.14)	0.519	1.24(1.00-1.52)	0.046	1.22(1.00-1.47)	0.046	1.26(1.04–1.53)	0.019	1.56(1.25–1.93)	<0.001
Q4	1.59(1.29–1.97)	<0.001	1.63(1.32–2.01)	<0.001	2.81(2.21–3.56)	<0.001	3.21(2.61–3.95)	<0.001	3.33(2.70–4.11)	<0.001	5.33(4.19–6.78)	<0.001

*OR* odds ratio, *CI* confidence interval

Model 1: adjusted for age, and sex

Model 2: adjusted for variables in Model 1 + marital status, education level, personal average annual income, drinking

Model 3: adjusted for variables in the Model 2 + BMI, WC, SBP, DBP, TC, LDL-C, and HDL-C.

### Subgroup analysis

**Fig. 2:**
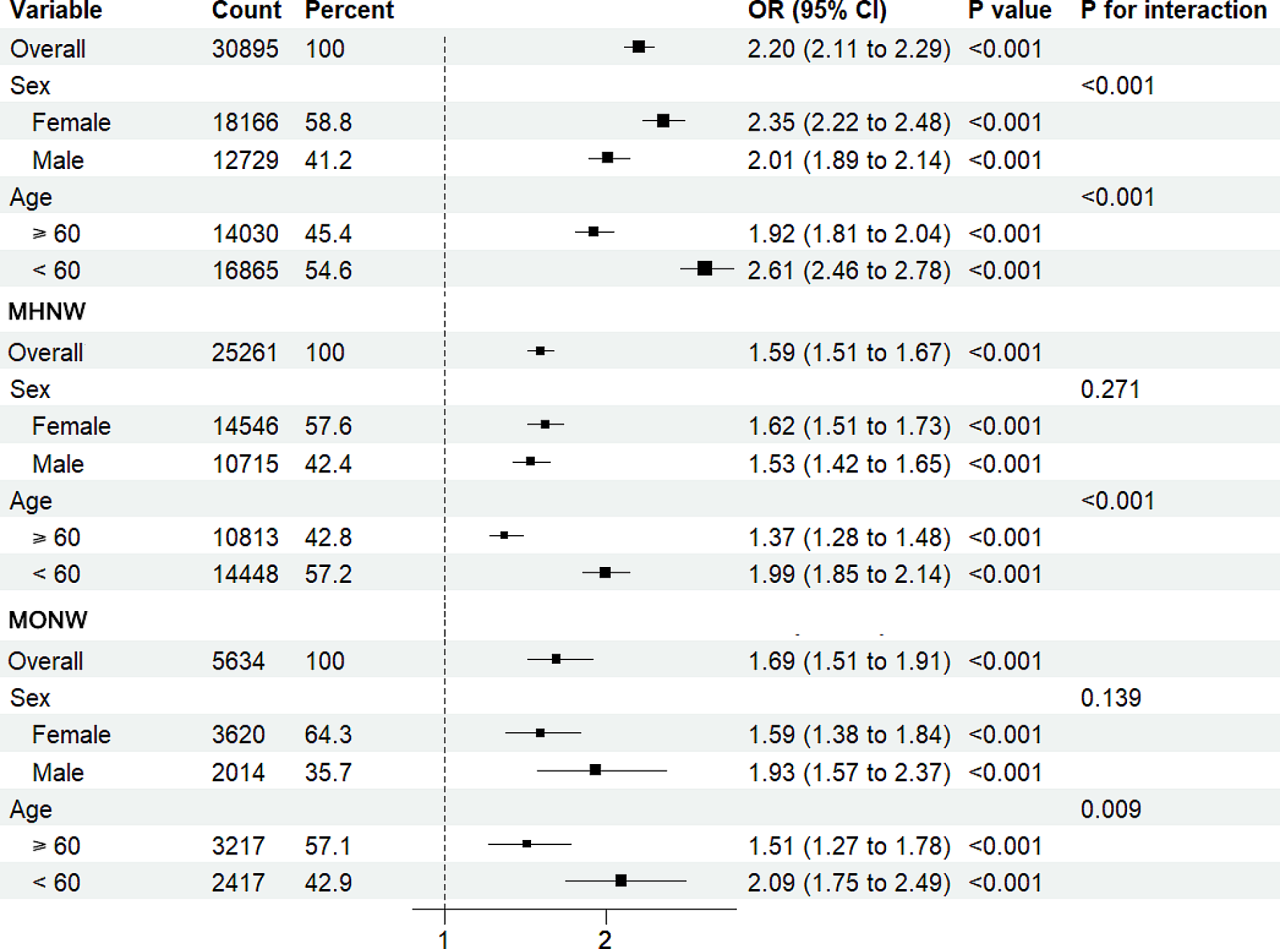
Subgroup analysis for the risk of stroke by the TyG index. Adjusted for survey age, sex, marital status, education level, personal annual income, and drinking, except for the variable used in each stratified analysis. OR odds ratio, CI confidence interval

Figure 2 shows the results of subgroup analysis. There was a significant interaction in the age and sex subgroups in the overall population (*P* for interaction <0.001). A high TyG index in females (OR, 2.35; 95% CI, 2.22–2.48, *P* < 0.001) was more significantly associated with an increased risk of stroke than in males (OR, 2.01; 95% CI, 1.89–2.14). Among people under 60 years old (OR, 2.61; 95% CI, 2.46–2.78, *P* < 0.001), a higher TyG index was more significantly associated with a higher risk of stroke than in people aged 60 years or older (OR, 1.92; 95% CI, 1.81–2.04, *P* < 0.001). However, there was no significant interaction between sex subgroups in the MHNW (*P* for interaction = 0.271) and MONW (*P* for interaction = 0.139) populations. In these two populations, among participants under 60 years old, a higher TyG index was more significantly associated with an increased risk of stroke compared to those aged 60 or older, and this risk association was more pronounced in the MONW population (OR, 2.09; 95% CI, 1.75–2.49, *P* < 0.001).

**Fig. 3:**
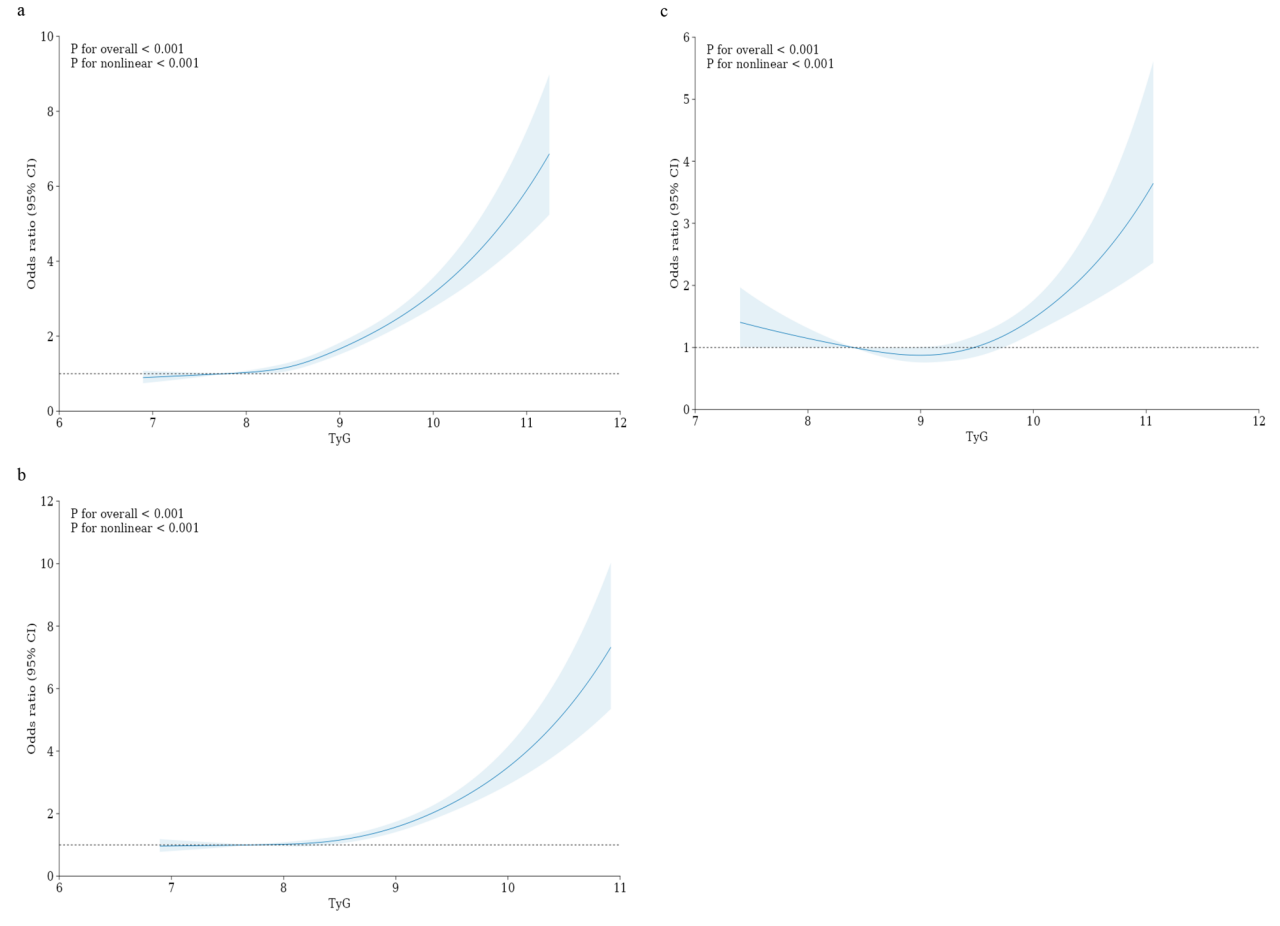
The associations of TyG index with stroke risk (**a**) overall, (**b**) MHNW, and (**c**) MONW. Data were fitted using the linear regression models of the restricted cubic spline with 4 knots at 5th, 35th, 65th, and 95th percentiles of TyG index. TyG index triglyceride-glucose index, CI confidence interval. The reference point was the median of the TyG index. The solid line represented point estimation on the association of TyG index with stroke risk, and the shaded portion represented 95% CI estimation. Covariates in the model included age, sex, marital status, education level, personal average annual income, drinking, BMI, WC, SBP, DBP, TC, LDL-C, and HDL-C

The results of the restricted cubic spline curve are shown in Fig. 3. The TyG index and stroke risk were shown to be nonlinearly associated with the overall population and the MHNW/MONW population (non-linear *P*<0.001). In addition, in the MONW population, the RCS curve is U-shaped. According to the graph, there is a considerable reduction in the risk of stroke within a lower TyG index range. However, the risk of stroke gradually increases as the TyG index climbs up to a certain point. Specifically, when the TyG index is < 8.5 or > 9.5, the stroke risk is relatively high in the MONW population. When the TyG index is in the range of 8.5–9.5, it serves as a protective factor against stroke in the MONW population.

## Discussion

In this population-based study, it was discovered that the TyG index was significantly associated with the risk of stroke levels among normal-weight individuals. The association between the TyG index and stroke risk was significant in both MONW and MHNW individuals, with a more pronounced association observed in MONW individuals. These findings suggest that the TyG index is associated with stroke risk and may be useful in identifying and monitoring stroke risk in normal-weight individuals.

This study found that among individuals with normal-weight, those with higher TyG index were associated with an increased risk of moderate to high stroke risk, consistent with previous research findings [21, 22]. Previous research has concentrated on examining the correlation between the TyG index and clinical outcomes of stroke. However, this study classifies the population into three degrees of stroke risk-low, moderate, and high-using stroke scale scoring standards. Subsequently, it was evaluated whether the TyG index is a useful indication for assessing the risk of stroke levels in the context of this framework. Previous studies have established that the NHCSSPP-proposed Stroke Risk Assessment Scale indicates enhanced usability and reliability in comparison to the modified FSRP scale and Framingham Stroke Profile (FSP) [23].

Exploring reliable indicators for assessing stroke risk levels in normal-weight individuals will contribute to advancing personalized preventive treatment strategies. Prior studies have suggested that stroke individuals with high TyG index levels face a heightened mortality risk and a more unfavorable prognosis [24]. However, compared to individuals of normal weight, stroke patients who are overweight/obese have a higher survival rate [25]. In South Africa, normal-weight persons have a higher risk of all-cause mortality compared to those who are overweight or obese [26]. The possible explanation is that people with normal BMI tend to ignore their cardiometabolic concerns. There is evidence that by implementing population-based screening for stroke risk factors and comprehensive interventions, the incidence and recurrence of stroke can be reduced, which would lower disability and the socioeconomic burden caused by stroke [27]. Therefore, there is a need for metabolic risk screening in individuals with normal-weight. The findings of this study indicate that TyG is beneficial for identifying persons with a moderate to high risk of stroke within the normal-weight population. However, further evidence is needed to establish its efficacy as a useful indicator for stroke risk grading in other populations.

Within the normal-weight category, MONW persons are regarded as a group at high risk for CVD. More over 1/3 of Chinese individuals with normal-weight have mild to moderate cardiometabolic diseases, according to the available evidence [28]. Alternatively, a large study also discovered that people with metabolic abnormalities are more susceptible to CVD than those without metabolic abnormalities [29]. An almost 2-fold increase in stroke incidence was observed in MONW individuals compared to MHNW individuals, with a risk of 1.82 (95% CI: 1.59–2.07) [6]. Even among menopausal women, the risk of stroke is 3.61 (95% CI, 1.18–11.03) times greater in MONW individuals than in MHNW individuals [30]. This study found that the association between the TyG index and moderate-to-high stroke risk was significantly stronger in MONW individuals compared to MHNW individuals. Similar to earlier study findings, related research found a significant association between the TyG index and CVD risk factors such as hyperglycemia and hyperlipidemia in the MONW phenotype, demonstrating marked gender disparities [31]. The TyG index has been proven effective as an indicator of metabolic abnormalities even within the normal-weight range, demonstrating high sensitivity and specificity, particularly in detecting the MONW phenotype [31]. According to previous investigations, the ORs of being classified as MONW group increased in a stepwise fashion throughout the TyG index quartiles among normal-weight subjects [32]. These findings imply that the TyG index plays an increasing part in stroke pathology, underscoring the need to maintain stable low TyG index levels in stroke prevention.

Additionally, Further subgroup analyses stratified by age and sex revealed that the TyG index has a stronger association with moderate to high stroke risk in females and individuals under the age of 60. For females or relatively younger individuals, high levels of stroke risk may result from the interplay of various physiological and metabolic characteristics. For instance, fluctuations in the female hormone estrogen during physiological cycles could contribute, while middle-aged individuals might be undergoing hormonal level changes. These hormonal fluctuations could influence blood glucose homeostasis and lipid metabolism, leading to changes in the TyG index, and thereby affecting stroke risk [33]. Notably, subgroup analyses also found that the MONW population remained at a higher level of stroke risk, especially in males, and younger than 60 years. Despite the potential impact of sample size reductions in each subgroup, age, and gender may be major factors in stroke risk in the MONW population. However, since the results of subgroup analysis are provisional, it is essential to interpret the observed differences in associations between subgroups cautiously. Further research is required to confirm potential differences in age, gender, socioeconomic status, BMI, and other factors.

Population-based risk assessment and screening for stroke risk have important public health implications. Superficially, individuals with MONW may greatly benefit considerably from secondary prevention strategies for CVD. Nevertheless, it is important to recognize that persons with MONW are frequently disregarded or concealed throughout the process of screening and detecting pertinent risk variables, and thus receive less focus in health care. Given their high risk of developing CVD [14], this population should receive increased medical attention akin to that of the obese population. Therefore, researching and introducing appropriate, cost-effective screening tests with enhanced sensitivity is feasible for apparently healthy individuals. Due to the widespread availability and routine performance of TG and FBG measurements in primary healthcare settings, the TyG index is more widely used in clinical and epidemiological research. By incorporating the TyG index into clinical practice, primary healthcare practitioners can improve their capacity to detect stroke risk in seemingly healthy patients, thereby adopting more logical treatment or preventative measures.

The study examined stroke risk levels and subgroup analysis of different metabolic states of normal-weight based on the characteristics of participants. Several limitations need to be explained. First, cross-sectional designs cannot determine causality, and more comprehensive longitudinal investigations are required to validate the findings. Second, this study included only middle-aged and older, normal-weight participants from China, and caution should be exercised when extrapolating results to other populations. Thirdly, this study primarily focused on assessing stroke risk using the Stroke Risk Assessment Scale rather than directly analyzing actual stroke incidence, which may affect the universality of the research results in practical clinical application. Future research should include long-term follow-up of actual stroke incidence to further validate and extend the findings of this study.

## Conclusion

This study suggests that the higher the TyG index in normal-weight individuals, the higher the stroke risk level, particularly females and individuals below the age of 60. Given its straightforward measurement and effective functionality, the TyG index may be a useful indicator for identifying individuals at risk for stroke in normal-weight populations.

### Electronic supplementary material

Below is the link to the electronic supplementary material.

Supplementary Material 1

## Data Availability

No datasets were generated or analysed during the current study.

## Abbreviations

TyG: Triglyceride glucose
NHCSSPP: The National Health Commission of Stroke Screening and Prevention Projects
IR: Insulin resistance
HOMA-IR: Homeostasis Model Assessment
CVD: Cardiovascular disease
T2DM: Type 2 diabetes mellitus
BMI: Body mass index
MONW: Metabolically obese normal-weight
MHNW: Metabolically healthy with normal-weight
FBG: Fasting plasma glucose
WC: Waist circumference
BP: Blood pressure
SBP: Systolic blood pressure
DBP: Diastolic blood pressure
TG: Triglyceride
TG: Total cholesterol
LDL-C: Low-density lipoprotein cholesterol
HDL-C: High-density lipoprotein cholesterol
