# Supplementary material for: Association between the triglyceride-glucose (TyG) index and stroke risk in Chinese normal-weight adults: a population-based study
Source: Diabetol Metab Syndr. 2024 Jul 25;16:176. doi: 10.1186/s13098-024-01421-w (PMC11270772; doi:10.1186/s13098-024-01421-w)
Supplement: Supplementary file 1 — Supplementary Material 1 [file 13098_2024_1421_MOESM1_ESM.docx]

**Supplementary Table**

**Supplementary Table 1.** Demographic data analysis of included and excluded subjects

| **Characteristics** | **Included**  **(n=30,895)** | **Excluded**  **(n=28,864)** | ***P-*value** |
| --- | --- | --- | --- |
| Range of TyG index | 6.09-12.63 | 5.33-12.19 |  |
| Age(years) | 59.48±10.87 | 60.61(10.60) | ＜0.001 |
| Male, n (%) | 12729(41.20) | 13751(47.64) | ＜0.001 |
| High school and above, n (%) | 10553(34.16) | 9755(33.80) | 0.295 |
| Married, n (%) | 29220(94.58) | 27244(94.39) | 0.307 |
| Personal average annual income ≥20000 RMB, n (%) | 17102(55.36) | 16784(58.15) | ＜0.001 |
| Current smoker, n (%) | 3480(11.26) | 4196(14.54) | ＜0.001 |
| Current drinker, n (%) | 4370(14.14) | 6267(21.71) | ＜0.001 |
| Physical activity, n (%) | 20833(67.43) | 18015(62.41) | ＜0.001 |
| Atrial fibrillation, n (%) | 283(0.92%) | 324(1.12) | 0.012 |
| Family history of stroke, n (%) | 2321(7.51%) | 2542(8.81) | ＜0.001 |
| Previous stroke history, n (%) | 749(2.42%) | 1188(4.12) | ＜0.001 |
| Previous TIA history, n (%) | 391(1.27%) | 386(1.34) | 0.439 |
| BMI, kg/m^2^ | 21.97±1.34 | 26.11±3.23 | ＜0.001 |
| TyG index | 8.68±0.58 | 8.81(0.58) | ＜0.001 |
| WC, cm | 79(74-83.00) | 86.26(12.25) | ＜0.001 |
| SBP, mmHg | 125.5(117.5-135) | 130.5(122-142) | ＜0.001 |
| DBP, mmHg | 77.5(72-82.5) | 80(75-86) | ＜0.001 |
| TC, mmol/L | 4.60(4.00-5.34) | 4.64(4.04-5.34) | 0.032 |
| TG, mmol/L | 1.37(1.00-1.89) | 1.52(1.14-2.09) | ＜0.001 |
| FBG, mmol/L | 5.08(4.56-5.79) | 5.20(4.61-6.00) | ＜0.001 |
| LDL-C, mmol/L | 2.57(2.09-3.16) | 2.59(2.08-3.20) | 0.435 |
| HDL-C, mmol/L | 1.40(1.17-1.69) | 1.30(1.10-1.56) | ＜0.001 |

*Abbreviations:* *TIA*, transient ischemic attack; *BMI*, body mass index; *WC*, waist circumference; *SBP*, systolic blood pressure; *DBP*, diastolic blood pressure; *TC*, total cholesterol; *TyG*, triglyceride glucose; *FBG*, fasting blood glucose; *HDL-C*, high-density lipoprotein cholesterol; *LDL-C*, low-density lipoprotein cholesterol.
